# EEG and EMG dataset for the detection of errors introduced by an active orthosis device

**DOI:** 10.3389/fnhum.2024.1304311

**Published:** 2024-01-22

**Authors:** Niklas Kueper, Kartik Chari, Judith Bütefür, Julia Habenicht, Tobias Rossol, Su Kyoung Kim, Marc Tabie, Frank Kirchner, Elsa Andrea Kirchner

**Affiliations:** ^1^Robotics Innovation Centre, German Research Centre for Artificial Intelligence (DFKI), Bremen, Germany; ^2^Institute of Medical Technology Systems, University of Duisburg-Essen, Duisburg, Germany; ^3^Robotics Lab, University of Bremen, Bremen, Germany

**Keywords:** EEG, EMG, orthosis, dataset, error-related potential (ErrP), event-related potential (ERP), human-robot interaction (HRI), brain computer interface (BCI)

## 1 Introduction

Exoskeletons and orthoses are frequently used to facilitate limb movements in humans with motor impairments as they can integrate classical therapy approaches such as mirror therapy (Kirchner et al., 2013; Kirchner and Bütefür, 2022) using the electroencephalogram (EEG) signals. In addition to triggering exoskeleton assistance, EEG can also be used to infer movement intentions (Kirchner and Bütefür, 2022) which has been shown to be crucial for successful neuro-rehabilitation (Noda et al., 2012; Hortal et al., 2015). Furthermore, EEG can also be used to deduce the subjective correctness of the behavior of a robot that the human observes or interacts with, as demonstrated in several works by Iturrate et al. (2015) and Kim et al. (2017, 2020).

To validate the correctness of an assistive device, it is important to gain a deeper understanding of the level of support felt by the patients. Specifically, it is essential to assess whether the patients felt the mistakes made by the robotic assistive system. For some assistive devices, support can be visually observable, and subjective correctness can be validated and adapted based on the ErrP detected from the EEG signals (Batzianoulis et al., 2020). However, for robots worn by the patients, such as active exoskeletons or active orthoses (Kirchner and Bütefür, 2022), the patients may not see the incorrect behavior but feel it. Therefore, investigating whether tactile detection of incorrect behavior in an exoskeleton or orthosis evokes similar event-related potentials (ERPs) as visually observed behavior is of interest. This information can be used to correct the incorrect behavior perceived by the patient [for initial results of this published dataset and further discussion on utilizing different modalities for transferring error information, see Kim and Kirchner (2023)].

In EEG studies, the so-called error-related potential (ErrP) is evoked when erroneous behavior is observed (Iturrate et al., 2010; Kim and Kirchner, 2013), feedback indicating erroneous events is received (Holroyd and Coles, 2002) or when an error occurs during interaction (Kim et al., 2017). A comprehensive review of this is available in Chavarriaga et al. (2014). Moreover, inferring errors from EEG by detecting ErrPs is challenging because it requires asynchronous classification of relevant patterns (Kim et al., 2023). This asynchronous classification often results in numerous false positives due to long interaction times with the system or extended task execution times (Omedes et al., 2015; Spler and Niethammer, 2015; Lopes-Dias et al., 2021). In most studies, visual stimuli were used to evoke error-related potentials (ErrPs) (e.g., van Schie et al., 2004). While some studies used visuo-tactile stimuli to indicate upcoming events, the ErrPs were still evoked by the visual recognition of errors (e.g., Schiatti et al., 2018). To our knowledge, there are no studies in which tactile stimuli were used to recognize errors and evoke an ErrP. Based on the available literature, we identify this as a gap in the research on error-related activity. In addition to the visual modality, other modalities like tactile feedback, should be subject to closer investigation regarding their impact on brain activity.

In this work, we introduced easily recognizable force direction errors, opposing the planned body movements. The confirmation that errors have been recognized was provided by the subjects by pressing an air-filled ball. As it was likely that the subjects would react reflexively to these errors, other biosignals such as the electromyogram (EMG) were also recorded to better understand error processing as a whole. This recorded EMG data would also help us better understand what complexity in task, response, or interaction errors is required to elicit ErrPs.

Moreover, there is a need for more in-depth exploration to continuously classify error-related activities (Kim et al., 2023) and distinguish partially overlapping EEG activities. However, there is a particular shortage of openly accessible data that would enable a larger research community to contribute. The requirement of using robots to introduce tactile stimuli limits the number of research groups that could conduct research on these problems. To address this issue, we recorded a dataset of 8 subjects (10 sets per subject) wearing an active orthosis device that introduces simple errors in its behavior. In order to allow other groups to easily replicate and extend our work, details about the mechanical and electrical aspects of the simple elbow orthosis have been provided. Additionally, the experimental procedure has also been described in detail, including relevant information about the error complexity, subject instructions, and whether subjects should respond explicitly to errors.

We hope that this first open-access dataset of EMG an EEG data recorded during the processing of errors of tactile modality will enable broader research on how assistive technology can be improved by using EEG and EMG data to provide more natural and individualized support for activities of daily living. Such support is very important for rehabilitation purposes (Kornhaber et al., 2018).

The rest of the paper is structured as follows. Section 2 provides detailed information about the experimental design and methods used to record the dataset. It also describes the data format and the folder structure for a better understanding of the dataset. Section 3 presents a preliminary quality analysis of the recorded data in the form of response-time analysis and event-related potential analysis. Finally, in Section 4, we provide an overview of the conducted experiment and discuss future possibilities. A more comprehensive description of the hardware used in our setup is provided in the Supplementary material.

## 2 Methods and experimental design

This section provides information about the experimental design including details about subjects' informed consent, experimental setup and procedure, methods used for data acquisition, and the formatting of the recorded dataset.

### 2.1 Participants

Eight healthy subjects (four male and four female; average age 21.8 ± 2.4 years; right-handed; students) voluntarily participated in the study. A few days prior to the experiment, subjects received an introduction and underwent preliminary testing at the lab, which included checking the orthosis's fit and measurement of head circumference to determine their EEG cap size. All the subjects provided their written informed consent and were told they could stop the experiment at any time without consequences. Each experiment lasted for 4.9 ± 0.6 hours on average per subject on the same day at a stretch and all the subjects received a monetary compensation of 10€ per hour.

### 2.2 Experimental setup and procedure

An overview of all the protocols followed throughout the experiment is provided in this section.

#### 2.2.1 Subject preparation

**Figure 1:**
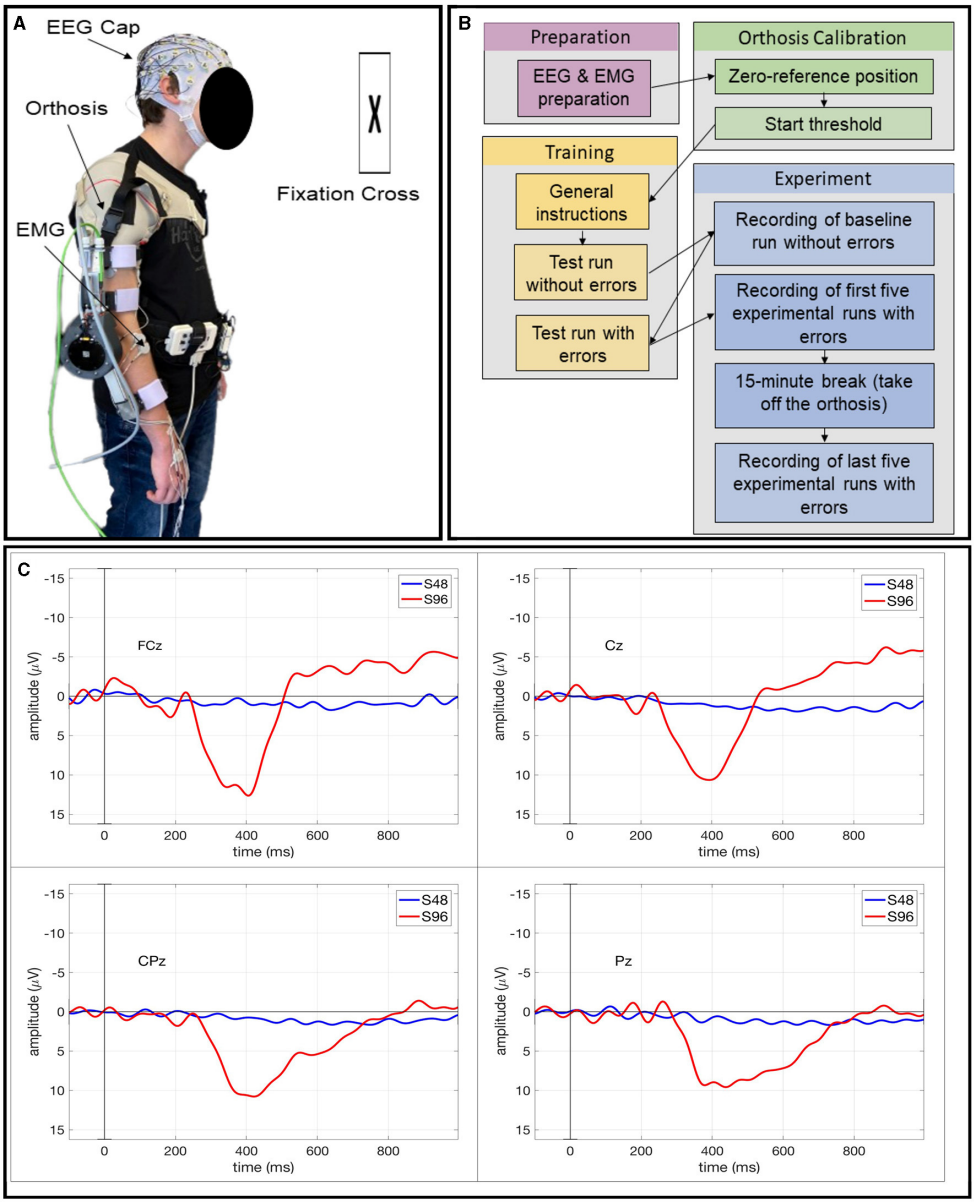
**(A)** Subject prepared with EEG and EMG electrodes wearing the orthosis on their right arm. **(B)** Visualization of the different steps in the experimental procedure. **(C)** ERPs averaged over all epochs within each event type: correct event (S48) and incorrect event (S96) for Subject AQ59D.

Before the start of the experiment, each subject was prepared with a 64-channel EEG system and an eight-channel EMG system (see Sections 2.3.1, 2.3.2 for details). Additionally, they were fitted with an active orthosis (see Section 1 of the Supplementary material) on their right arm as shown in Figure 1A, and held a small air-filled ball in their left hand (see Section 2 of the Supplementary material). To trigger support from the orthosis, the subjects were required to express their intention to move by applying a torque greater than the start threshold in the movement direction. This torque, applied at the forearm interface around the wrist (see Figure 1A and Supplementary Figure 1) induced a change in the elbow motor current which was then converted into the corresponding torque value.

The torque threshold varied among subjects, depending on their strength and the weight of their arm. It was ensured that the start thresholds were large enough to prevent unintended starts (refer to Section 3 of the Supplementary material). After indicating their intention to move, the subjects were instructed to ease their arm muscles as the orthosis took control of the movement and applied adequate torque at the elbow joint. A comprehensive list of the different thresholds for each subject can be found in Table 1A.

**Table 1:** (A, B): Orthosis parameters; (C, D): Results of response-time (RT) analysis based on the analysis from Kim and Kirchner (2023) (μ±σ : mean ± standard deviation).

**(A) Start thresholds for each subject**		
**Subject code**	**Start threshold (flexion)**	**Start threshold (extension)**
AQ59D	1.0 N-m	1.2 N-m
BY74D	0.8 N-m	1.2 N-m
AC17D	0.8 N-m	1.2 N-m
AW59D	0.7 N-m	1.2 N-m
AY63D	1.0 N-m	1.2 N-m
BS34D	1.2 N-m	1.4 N-m
AJ05D	1.0 N-m	1.2 N-m
AA56D	1.0 N-m	1.2 N-m
**(B) Operating parameters**		
**Parameters**		**Value**
Number of errors		6
Duration of errors		250 ms
Fully extended position		-10°
Fully flexed position		-90°
Maximum deviation		0.3°
Mean error position (Flexion)		-42°
Mean error position (Extension)		-58°
**(C) Median RT for each subject over 10 datasets**		
**Subject code**		**Response time**
AQ59D		0.67 s
BY74D		0.61 s
AC17D		0.83 s
AW59D		0.72 s
AY63D		0.67 s
BS34D		0.68 s
AJ05D		0.91 s
AA56D		0.89 s
μ±σ		0.75 ± 0.11
**(D) Median RT for each set over 8 subjects**		
**Dataset**		**Response time**
Set 1		0.72 s
Set 2		0.73 s
Set 3		0.70 s
Set 4		0.71 s
Set 5		0.70 s
Set 6		0.74 s
Set 7		0.80 s
Set 8		0.76 s
Set 9		0.68 s
Set 10		0.74 s
μ±σ		0.73 ± 0.03

#### 2.2.2 Experimental procedure

The experiment aimed at using tactile feedback to detect errors intentionally introduced during the flexion or extension movements of the active orthosis. Here, the term *error* refers to a momentary change in the direction of orthosis movement for a short duration of time (see Section 2.2.3 and Table 1B for more details). Furthermore, the term *movement trial* will be used to indicate a complete range of either flexion *or* extension movement.

The experiment was divided into three sessions per subject - a preliminary session, a training session and the main session. In the preliminary session, the subjects performed 30 movement trials (15 flexions and 15 extensions) with no errors to obtain a baseline. This was followed by a training session wherein the subjects got familiarized with how the errors felt and were also instructed to squeeze the ball in their left hand upon detecting an error. Ultimately, the main session consisted of 10 experimental runs each consisting of 30 movement trials (15 flexions and 15 extensions). Within each run, six errors were randomly introduced among the 30 movement trials (see Section 2.2.3 for more details).

Before each run, subjects were reminded to stand still to prevent motion artifacts in the EEG and EMG data. They were also instructed to fixate their eyes upon a black cross against the white wall in front of them to minimize eye artifacts in the EEG data. The experimental run began only after subjects heard a start phrase from the experimenter. It was also brought to their notice that if, for some reason, they felt an error but forgot to squeeze the ball, they shall just proceed with the run. The subjects were not notified of missed errors during the experiment. At the end of a run, the orthosis motor automatically disabled itself, and the subjects were informed via a stop phrase. After five runs, a 15-min break allowed subjects to relax and remove the orthosis. A visual summary of the whole experimental procedure is provided in Figure 1B.

#### 2.2.3 Error introduction

As defined in Section 2.2.2, the term *error* refers to a momentary change in the direction of orthosis movement for a short duration of time (here, 250 ms, as mentioned in Table 1B). All 30 movement trials were sequentially numbered from 1 to 30. Out of these, six were randomly selected for the introduction of errors near the *Mean error position* within the movement trial. The error position varied for flexion and extension with a *Maximum deviation* of 0.3° from the *Mean error position* as mentioned in Table 1B.

Furthermore, before randomly selecting the error trials, the following conditions were considered:

- Errors were not introduced in the 1^*st*^ and 2^*nd*^ movement trials.
- Errors were not introduced in two consecutive movement trials.

In practice, if the orthosis was executing flexion before the introduction of the error, it would transition into an extension for the specified duration of error (see Table 1B) and then resume flexion until the end of the trial, and vice-versa.

### 2.3 Data acquisition

This section provides detailed information about the methods used for recording the EEG and EMG data. Additionally, it also describes the process of synchronization of these two types of data.

#### 2.3.1 EEG recording

The EEG data were recorded using the 64-channel LiveAmp64 system from Brain Products GmbH^1^ and an ActiCap slim electrode system^2^ with an extended 10–20 layout. The reference electrode was placed at *FCz* and the GND at *AFz* electrode positions.

Great efforts were made to record high-quality EEG data and minimize the noise in the data by keeping the impedances of all 64 electrodes below a threshold of 5 kΩ. This impedance check was performed both prior to and after each experimental run. The EEG data were recorded using the *Recorder* software^3^ (version 1.25.0001) from Brain Products GmbH. The sampling rate was 500 Hz and the measurement system used hardware filters that limited the bandwidth of the data to a passband of 0.0 Hz–131.0 Hz.

#### 2.3.2 EMG recording

To record bipolar EMG data, the ANT mini eego amplifier^4^ was used. The EMG data were recorded with a sampling rate of 1000 Hz using an adapted eego SDK^5^ for Python. Eight channels were used, each measuring the muscle activity of the following muscles on both the arms:

- M. biceps brachii
- M. triceps brachii lateral
- M. triceps brachii long head
- M. flexor digitorum superficialis

Before placing the electrodes, the skin was prepared with Isopropyl alcohol (70% V/V). The electrodes were placed on the muscle belly in accordance with the SENIAM guidelines (Hermens et al., 2000).

#### 2.3.3 Synchronization of EMG and EEG data

To synchronize EEG and EMG data for offline analysis, an *Event Trigger* board (see Section 2 of the Supplementary material) along with the Sensor & Trigger extension from the EEG system were used. This setup enabled recording the start and end time points of the EMG data within the EEG data. Thus, the EEG system was used as the main device to enable the alignment of both data streams with respect to each other. Despite the EEG data recordings starting before EMG, the marked events served as reference points to align both data streams. However, it has to be noted that both data streams (EEG and EMG) were recorded with different measurement devices with different sampling rates (see Sections 2.3.1, 2.3.2). Nevertheless, with this approach, an average time difference below 8.5 ms between both data streams was achieved after evaluating the synchronicity for all recorded data sets. This result was obtained by comparing the amount of recorded data between the marked events in both data streams and converting the difference into time (in ms). Please refer to Section 2.4.3 for the specification of the marked events in the EEG data.

### 2.4 Dataset and format

This section describes the data format, along with detailed information about the dataset and the recorded events.

#### 2.4.1 Data format

The recorded EEG data follows the BrainVision Core Data Format 1.0, consisting of a binary data file (*.eeg*), a header file (*.vhdr*), and a marker file (*.vmrk*).^6^ For ease of use, the data can be exported into the widely adopted BIDS format as described in Gorgolewski et al. (2016). Furthermore, for data analysis, processing and classification, two popular options are available - MNE (Python)^7^ and EEGLAB (MATLAB).^8^ In contrast, the EMG data is stored in the *.txt* format, where each column represents a separate EMG channel.

#### 2.4.2 Dataset description

In this section, the dataset's folder structure is explained along with the convention used for naming the files.

##### 2.4.2.1 Folder structure

This section describes the hierarchical folder structure of the recorded dataset. At the highest level, there are three folders, namely *EEG, EMG*, and *Metadata*. The *Metadata* folder contains a *.txt* file for each subject, segregated by a unique code, which consists of meta-information about the subject as well as the measurement sets. In addition to these files, there is also a *short_description.txt* file with some general information about the whole study.

Furthermore, within each of the modality folders (*EEG* or *EMG*), there is an additional level of folders segregated by subject codes. Inside the *EEG* folder, each subject sub-folder is further divided into two sub-folders namely *data* and *imp*. The *data* folder consists of the actual measurement files as described in Section 2.4.1. In total, there is one baseline set without any errors stored inside another sub-folder named *baseline_without_error* and 10 sets with deliberate errors introduced. Each header file (*.vhdr*) also contains the impedance values of every electrode before the set. Conversely, each header file inside the *imp* folder contains impedance values after each set. All in all, all impedance values, before and after the set, are available within the header files (*.vhdr*). It has to be noted that, for some subjects, an additional set was recorded for redundancy and included in this dataset under a sub-folder named *additional sets*. For more detailed information, please refer to the Metadata readme files included within the dataset.

##### 2.4.2.2 Naming convention for data files

A consistent naming convention was followed for all our files, dividing the filename into five segments. The first segment denotes the date of data acquisition in *yyyymmdd* format (e.g., 20230424), followed by the subject code (e.g., AC17D). The third segment includes the experiment identifier, in this case, *orthosisErrorIjcai*, followed by *multi* distinguishing it from previously conducted experiments. For baseline runs, the suffix *baseline_set* along with the set number (e.g., *1* or *2*) was appended, while for experimental runs with errors, only the run number was appended at the end (e.g., *set5*). For instance, a filename would look like *20230424_AC17D_orthosisErrorIjcai_multi_set1.txt*. It is important to note that the term *set* was used to represent the data files associated with the corresponding experimental run.

#### 2.4.3 Recorded events

The events occurring during the run were systematically recorded and stored in marker files (*.vmrk*) to enable offline tracking. These marker files are located within the *data* sub-folder of each subject inside the *EEG* folder (see Section 2.4.2 for data structure). The first event (after the start of a run) was named *S1* and marked the start of the EMG recording for synchronization purposes (see Section 2.3.3 for detailed information). The event *S1* also occurred at the end of the EMG measurement. The next recorded event was *S64* which marked the start of flexion movement. Similarly, the start of an extension movement was marked by the event *S32*. In order to mark a trial without errors, the event *S48* was added around the *Mean error position* as mentioned in Table 1B. The event *S96* occurred as soon as an error was introduced in the trial. Additionally, if the subject squeezed the ball, the event *S80* was recorded in the marker file.

## 3 Analysis of data quality

In the following, we performed some basic analysis of the recorded data to validate its data quality and briefly describe the evoked event-related responses in EEG. Furthermore, to ensure data quality, invalid measurement sets were categorized based on the reason of exclusion and subsequently removed from the data repository. The different exclusion categories are as follows:

- *Subject behavior*: activities that the subjects were informed against performing (e.g.,: playing with the air-filled ball during the experiment).
- *Technical issues*: issues that affected the functioning of the orthosis (e.g.,: safety shutdown due to maximum current/torque limit reach, issues with recording events, CAN communication issue resulting in failure to start the orthosis device, etc.).
- *Artifacts and noise*: detected in the EEG during live visualization (e.g.,: excessive teeth clenching or head movements).

The excluded sets of all subjects are listed below in the form of *subject code* and *set number* followed by the exclusion category. The excluded sets are as follows:

- *AA56D, set8*: Technical Issues.
- *AC17D, baseline_set1*: Artifacts and Noise.
- *AJ05D, baseline_set1*: Technical Issues.
- *AJ05D, set8*: Artifacts and Noise.
- *AJ05D, set9*: Artifacts and Noise.
- *AQ59D, set1*: Technical Issues.
- *AW59D, set1*: Artifacts and Noise.
- *AY63D, baseline_set1*: Subject Behavior.

Each of the rejected sets was excluded and supplemented by an additional measurement set (as mentioned in Section 2.4.2.1). While efforts were made to ensure high data quality, we observed high-frequency noise in some EEG channels, likely induced when subjects accidentally had contact with the unisolated parts of the orthosis. Additionally, a 50 Hz noise was also observed in the EMG data. Notably, for subject AQ59D, measurements were taken for the muscle M. extensor digitorum instead of M. flexor digitorum superficialis and for this subject, no alcohol was used for skin preparation. Furthermore, for subject BY74D, EMG data for set 3, set 4 and set 5 were not recorded due to technical problems.

### 3.1 Behavioral analysis

For response-time analysis, we analyzed the time taken by each subject to respond to error events. As mentioned above, the subjects were instructed to squeeze an air-filled ball after recognizing an error. The time between the error event (S96) and the response to the event (S80) was calculated for all events.

According to the experimental design, we expected a total of 480 responses to error events (6 error events × 10 datasets × 8 subjects = 480 error events). However, we found 9 false negative cases (i.e., the ball was not squeezed, after an error event occurred) and 5 false positive cases (i.e., the ball was squeezed, although the error event did not occur). Hence, a total of 471 error event-response pairs (480 error events - 9 false negatives) were used to compute the response times. We obtained a median value of 0.72 s over 471 error event-response pairs.

We also performed two additional analyses. First, we calculated the response time averaged over all 10 sets for each subject (see Table 1C). Furthermore, we also calculated the response time averaged over all 8 subjects for each set (see Table 1D).

### 3.2 Event related potentials analysis

For ERP analysis, we analyzed the EEG data of one subject using EEGLAB (see text footnote 8). We preprocessed the data as follows. The raw EEGs were downsampled to 250 Hz, re-referenced to an average reference, and filtered between 0.1 Hz and 15 Hz. The *FCz* channel, used as a reference in the EEG recording, was recalculated as an EEG channel for ERP analysis. After preprocessing, eye artifacts were rejected by visual inspections. Hence, EEG data without ocular artifacts was used for EEG segmentation. This EEG data was segmented into epochs from 0.1 s to 1 s after each event type (correct/incorrect). Epochs were averaged within each event type with a baseline correction (-0.1 s until stimulus onset).

For averaging epochs, we only used the error events with correct responses i.e., true positive cases (the ball was squeezed when error events occurred). Figure 1C shows the ERPs averaged over all epochs for each event type (S48: correct event, S96: incorrect event) for Subject AQ59D. The ERP morphology, i.e., the shape and distribution on the scalp suggests that introducing errors elicits a P300 component, specifically a P3b (Polich, 2007) component. This may be elicited by infrequently occurring odd events to which subjects respond, i.e., task-relevant events (Kirchner et al., 2016). In the case of this subject, we could not observe a strongly expressed error-related potential (ErrP) pattern, which is usually evoked by the recognition of errors since there was a strong overlay by a P300 potential. However, the overall shape of the evoked ERPs was similar to the shape found in Chavarriaga et al. (2012). We observed a first negativity around 250 ms followed by a positivity between 300 ms and 500 ms and a further negativity around 600 ms. Results for all subjects and grand average ERP analysis for this dataset can be found in Kim and Kirchner (2023).

## 4 Conclusion

We presented and described an open-access dataset containing EEG and EMG data from eight subjects assisted by an active orthosis device in moving their right arm. Behavioral analysis showed the subjects' excellent recognition of errors which were a momentary change in the direction of movement of the orthosis for a short duration of time. The errors were simple and easily tactilely detectable. The appearance of an ERP in the form of *P3b* indicates that the subjects recognized the erroneous events as odd events. The absence of a strong ErrP in the EEG during error introduction may be attributed to the strong overlay with the elicited P300 potential and the simplicity of the error. These conclusions are very preliminary based on an analysis of only one subject. Our further analysis (Kim and Kirchner, 2023) showed some subjects with strong ErrP, weak P300, and a clearly visible ErrP in the grand average. We hope that the provided dataset and detailed information about the experimental setup will allow its replication enabling the research community to systematically investigate the relationship between odd-event detection and erroneous event evaluation evoked in the brain. A deeper understanding of this relationship could help in the further development of approaches that could allow automatic adaptation of an assistive device to a subject's individual needs.

## Data availability statement

The datasets presented in this study can be found in online repositories: https://doi.org/10.5281/zenodo.8345429. The names of the repository/repositories and accession number(s) can be found in the article/Supplementary material.

## Ethics statement

The studies involving humans were approved by Ethics Committee of the Department of Computer Science and Applied Cognitive Science of the Faculty of Engineering at the University of Duisburg-Essen. The studies were conducted in accordance with the local legislation and institutional requirements. The participants provided their written informed consent to participate in this study. Written informed consent was obtained from the individual(s) for the publication of any potentially identifiable images or data included in this article.

## Author contributions

NK: Data curation, Investigation, Methodology, Resources, Software, Validation, Writing – original draft, Writing – review & editing. KC: Data curation, Investigation, Methodology, Resources, Software, Validation, Visualization, Writing – original draft, Writing – review & editing. JB: Data curation, Investigation, Methodology, Resources, Visualization, Writing – original draft, Writing – review & editing. JH: Data curation, Investigation, Methodology, Resources, Writing – original draft, Writing – review & editing. TR: Investigation, Resources, Validation, Writing – original draft, Writing – review & editing. SK: Conceptualization, Formal analysis, Methodology, Validation, Visualization, Writing – original draft, Writing – review & editing. MT: Resources, Software, Writing – review & editing. FK: Funding acquisition, Project administration, Supervision, Writing – review & editing. EK: Conceptualization, Funding acquisition, Methodology, Project administration, Resources, Supervision, Writing – original draft, Writing – review & editing.

## Footnotes

^1^ https://www.brainproducts.com/solutions/liveamp/

^2^ https://www.brainproducts.com/solutions/acticap/

^3^ https://www.brainproducts.com/downloads/recorder/

^4^ https://www.ant-neuro.com/products/eego_8

^5^ https://gitlab.com/smeeze/eego-sdk-pybind11/-/tree/0ace9b329b7cf5f6d1da5d387d0f2a5c07e87ee7

^6^ https://www.brainproducts.com/support-resources/brainvision-core-data-format-1-0/

^7^ https://mne.tools/stable/api/python_reference.html

^8^ https://sccn.ucsd.edu/eeglab/index.php
