# Supplementary material for: EEG and EMG dataset for the detection of errors introduced by an active orthosis device
Source: Front Hum Neurosci. 2024 Jan 22;18:1304311. doi: 10.3389/fnhum.2024.1304311 (PMC10839100; doi:10.3389/fnhum.2024.1304311)
Supplement: Supplementary file 1 [file Data_Sheet_1.pdf]

Supplementary Material

1 ACTIVE ORTHOSIS DEVICE

The orthosis device (**Figure S1**) was mainly built from off-the-shelf components and in-house manufactured parts (e.g. 3D printed or water jet cut) resulting in a simple and cost-effective design. The orthosis consists of an upper arm and a forearm structure connected and driven by an actuator. To compensate for the weight of the device and thereby enhance wearer comfort, the upper arm structure is attached to a strap that is sewn on a shoulder orthosis (see **Figure 1-(A)** from the paper).

To transmit forces to the wearer's arm, the elbow orthosis connects to the human body via an upper arm and a forearm interface. The cuffs consist of silicone pads cast on bent, water-jet-cut aluminum sheet parts. The sheet parts transmit the interaction forces between the orthosis and the wearer and can be deformed plastically to individual arm shapes. The silicone pads not only provide soft contact points to the human tissue but also friction between the arm and interfaces to keep the orthosis in place.

To adapt the device to different wearers, both upper arm and forearm structure lengths can be adjusted. While the upper arm length is fixed after adjustment, the forearm interface is attached to a slider. This prismatic joint adds a passive degree of freedom to the system that not only adjusts the forearm length but also compensates for misalignments between the axes of rotation of the elbow and orthosis. For further individual adjustment, the forearm interface can be inclined.

The actuator is a T-MOTOR AK80-6 with a rated torque of $6\,\mathrm{N \cdot m}$ ($12\,\mathrm{N \cdot m}$ peak). This drive belongs to the category of so-called quasi-direct drives or proprioceptive actuators (Wensing et al. (2017)). These comprise a high torque density motor combined with a low gear ratio transmission. Such actuators feature high intrinsic back-drivability and simultaneously allow accurate high-bandwidth force control. The quasi-direct drive actuation paradigm has been introduced in rehabilitation robotics throughout the last years, e.g., in Lv et al. (2018), Yu et al. (2020).

For communication with the AK80-6, we used a USB to CAN interface and the driver software of Shubham Vyas (2023). We operated the drive in position control mode using the onboard low-level controller. In addition to the software limits, a mechanical stop constrains the range of motion of the orthosis device from the extension hard limit (upper arm and forearm structure in parallel) to a flexion of $-130°$, for safety reasons. As we used the drive in position control mode only, we did not exploit the full potential of quasi-direct drive actuation in terms of transparent force control in this work. However, we are confident that this feature will prove useful in future use of the orthosis.

The device was powered by a laboratory power supply set to a voltage of $24\,\mathrm{V}$ and a current of up to $3\,\mathrm{A}$. An emergency stop was connected in series with the actuator in case of malfunctions, and a $2400\,\mu\mathrm{F}$, $100\,\mathrm{V}$ rated capacitor was added in parallel with the actuator for transient voltage suppression. Cable glands were used as strain reliefs to protect the data and power cable connectors from damage.

2 RESPONSE LISTENER AND EVENT TRIGGER BOARD

This section describes the hardware used to read explicit feedback and record important events during the experiment. The setup consisted of an *air-filled ball* that acted as an input device, a *Response Listener* and an *Event Trigger Board*. Whenever the subject felt an error in the orthosis device, they would press the air-filled ball in their left hand. The state of this ball was recorded by the *Response Listener*, a microcontroller that continuously read the analog inputs and converted them into digital inputs via a 16-bit analog-to-digital converter (ADC). It then transmitted the pin states, through a serial connection, to a Python script. This script then converted the pin states into

single-byte values and sent them serially to the *Event Trigger Board*, which is an ATmega328-based microcontroller board (Arduino Nano[1]).

Additionally, other events such as the error introduction, start of flexion or extension, and no-error movement trial were also sent as unique single-byte arrays to the *Event Trigger Board*. This board read the byte values serially and mapped them into transistor-transistor logic (TTL) output signals. These signals were eventually recorded into the electroencephalogram (EEG) marker files, as described in the *Recorded Events* section of the paper.

3 CALIBRATION SEQUENCE AND SAFETY MEASURES

For calibrating the zero-reference position of the orthosis, the subjects were asked to fully extend their arms to the hardware limit of the elbow motor. After this calibration, the orthosis would move to a more comfortable start position which was termed the *Fully Extended position* (see **Figure S2**). As a safety measure, the range of motion of the orthosis was restricted between the *Fully Extended position* and *Fully Flexed position* (both inclusive). On reaching the range limits, the elbow motor stopped automatically and held the position until the subjects crossed the start torque threshold. Moreover, another layer of safety was added by restricting the maximum current of the power supply and forcing motor torque limits in software.

Furthermore, the start thresholds for flexion and extension were calibrated individually. Initially, the threshold values were set to $1.0\,\mathrm{N}\cdot\mathrm{m}$ for flexion and $1.2\,\mathrm{N}\cdot\mathrm{m}$ for extension. To determine the appropriate individual threshold value for flexion, multiple flexion movement trials were performed by the subjects until they arrived at a force that felt neither too weak nor too strong. In contrast, to establish the optimal start threshold for each subject for extension, they were asked to rest their arms in the *Fully Flexed position* and the lowest value at which no unwanted extensions occurred was selected.

4 EXPERIMENT OVERVIEW AND EVENT RECORDING

This section describes the complete experiment along with all the events recorded by the EEG *Recorder* software with the help of block diagrams. As outlined in Section 2.2.2 of the data paper, each experiment comprised three sessions per subject. In the preliminary session, a baseline run was recorded as shown in **Figure S3** wherein the experiment start was denoted by the *S1* event. Following this, a *Relax Break* allowed subjects to calm down and minimize motion artifacts before proceeding to 30 movement trials (15 flexions and 15 extensions). The start of each flexion was marked by the *S64* event whereas *S32* marked extension starts. Furthermore, in error-free trials, the event *S48* was recorded around the *Mean error position* as mentioned in Table 1b of the data paper. The session concluded with the recording of another *S1* event.

The main session comprised of 10 experimental runs as shown in **Figure S4-(A)**. Each run consisted of 30 movement trials out of which intentional errors were randomly introduced in 6 trials, as per the conditions defined in Section 2.2.3 of the data paper. In case of the error trials, the event *S96* was recorded instead of *S48* as depicted in **Figure S4-(B)**.

[1] https://store.arduino.cc/products/arduino-nano

FIGURES

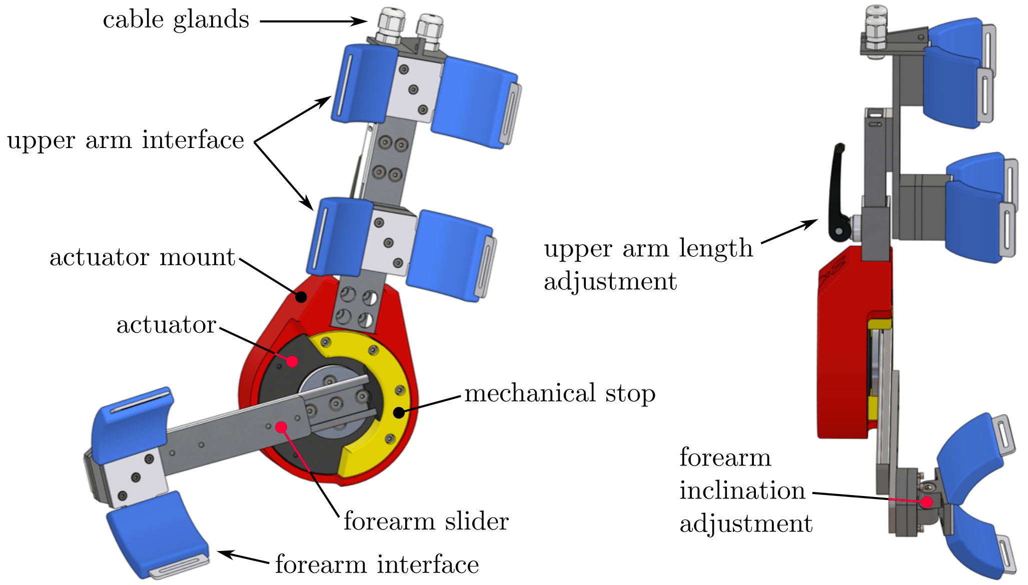

Figure S1: Computer Aided Design (CAD) model side and front view of the active orthosis device. The actuator mount and mechanical stop are colored for better visibility. The connection to the shoulder orthosis can be seen in Figure 1a of the paper.

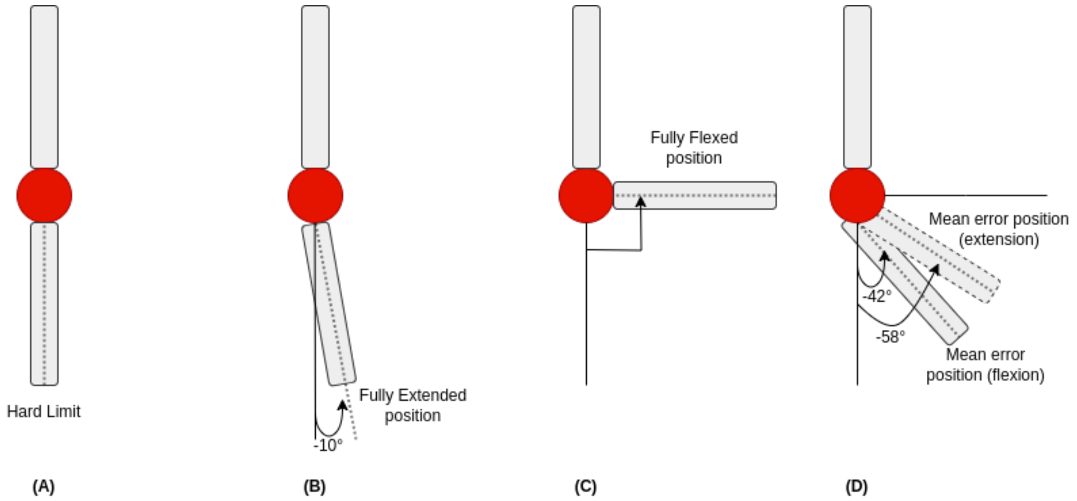

Figure S2: Different operating positions of the orthosis. **(A)** Zero-reference position for the experimental run. **(B)** Comfortable start position and extension limit during movement trial. **(C)** Flexion limit during movement trial. **(D)** Positions around which errors were introduced.

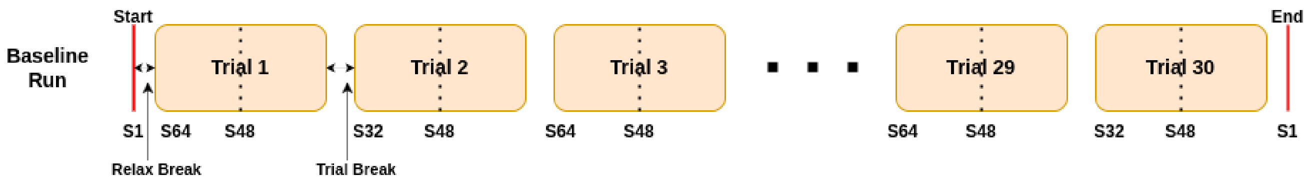

Figure S3: Block diagram depicting the baseline run consisting of 30 error-free movement trials

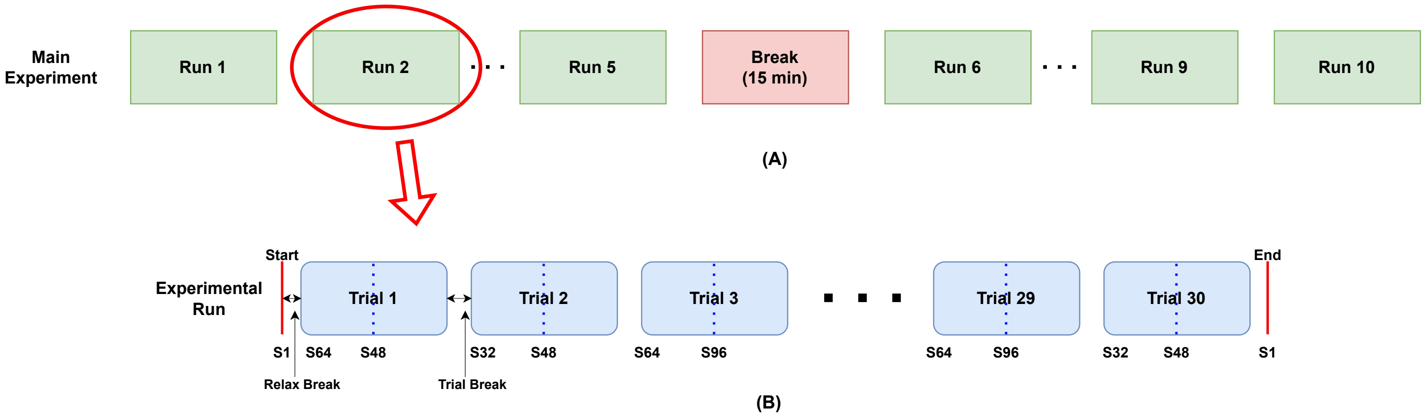

Figure S4: Block diagram for the main experiment. **(A)** Main experiment with 10 experimental runs with a 15-minute break after five runs. **(B)** One of the experimental runs is depicted here. Each experimental run consisted of 30 movement trials out of which errors were introduced in six randomly-selected trials.

REFERENCES

Lv, G., Zhu, H. & Gregg, R. D. (2018), 'On the design and control of highly backdrivable lower-limb exoskeletons: A discussion of past and ongoing work', *IEEE Control Systems Magazine* **38**(6), 88–113.

Shubham Vyas, Shivesh Kumar, J. B. (2023), 'dfki-ric-underactuated-lab/mini-cheetah-tmotor-python-can', *Zenodo* .
URL: https://doi.org/10.5281/zenodo.7671321

Wensing, P. M., Wang, A., Seok, S., Otten, D., Lang, J. & Kim, S. (2017), 'Proprioceptive actuator design in the mit cheetah: Impact mitigation and high-bandwidth physical interaction for dynamic legged robots', *IEEE Transactions on Robotics* **33**(3), 509–522.

Yu, S., Huang, T.-H., Yang, X., Jiao, C., Yang, J., Chen, Y., Yi, J. & Su, H. (2020), 'Quasi-direct drive actuation for a lightweight hip exoskeleton with high backdrivability and high bandwidth', *IEEE/ASME Transactions on Mechatronics* **25**(4), 1794–1802.
